# Depression and Anxiety in Hong Kong during COVID-19

**DOI:** 10.3390/ijerph17103740

**Published:** 2020-05-25

**Authors:** Edmond Pui Hang Choi, Bryant Pui Hung Hui, Eric Yuk Fai Wan

**Affiliations:** 1 School of Nursing, LKS Faculty of Medicine, The University of Hong Kong, Hong Kong, China; 2 Department of Sociology, Faculty of Social Sciences, The University of Hong Kong, Hong Kong, China; bryant09@hku.hk; 3 Department of Family Medicine and Primary Care, LKS Faculty of Medicine, The University of Hong Kong, Hong Kong, China; yfwan@hku.hk; 4 Department of Pharmacology and Pharmacy, LKS Faculty of Medicine, The University of Hong Kong, Hong Kong, China

**Keywords:** COVID-19, depression, anxiety, mental health, Hong Kong

## Abstract

It has been three months since the first confirmed case of coronavirus disease 2019 (COVID-19) in Hong Kong, and people now have a more complete picture of the extent of the pandemic. Therefore, it is time to evaluate the impacts of COVID-19 on mental health. The current population-based study aimed to evaluate the depression and anxiety of people in Hong Kong during the COVID-19 pandemic. Respondents were randomly recruited and asked to complete a structured questionnaire, including the patient health questionnaire-9 (PHQ-9), the generalized anxiety disorder-7 (GAD-7), the global rating of change scale and items related to COVID-19. Of the 500 respondents included in the study, 19% had depression (PHQ-9 score ≥ 10) and 14% had anxiety (GAD score ≥ 10). In addition, 25.4% reported that their mental health had deteriorated since the pandemic. Multiple logistic regression analysis found that not experiencing the SARS outbreak in 2003, being worried about being infected by COVID-19, being bothered by having not enough surgical masks and being bothered by not being able to work from home were associated with a poorer mental health status. Psychological support, such as brief, home-based psychological interventions, should be provided to citizens during the pandemic.

## 1. Introduction

The emergence and rapid increase in the number of cases of coronavirus disease 2019 (COVID-19), an infectious disease caused by severe acute respiratory syndrome coronavirus 2, pose complex challenges for global public health, research and medical communities [1]. Currently, COVID-19 is a public health emergency of international concern, as declared by the World Health Organization. As of 6 May 2020, there were more than 3.7 million confirmed cases of COVID-19 across more than 200 countries, areas and territories, including more than 250,100 deaths. 

The first confirmed case of COVID-19 in Hong Kong was announced on 23 January 2020. In response to the COVID-19 pandemic, the local government has adopted a variety of drastic public health measures, such as compulsory quarantines for people returning from abroad, work from home arrangements, school suspensions, and the shutdown of non-essential services, to mitigate the risks and impact of the disease. In addition, people in Hong Kong have stockpiled surgical masks, rice, toilet paper, and other goods. Such abrupt changes to daily life are risk factors that can substantially affect mental health. 

Some psychosocial stressors such as health threats to oneself and loved ones are associated with pandemics [2]. There are severe disruptions of routines, separation from family members and friends, shortages of daily necessities, salary deduction, social isolation, and school closure [2]. Psychosocial responses to infectious disease outbreaks are variable and can include feelings of anxiety or weakness, an overestimation of the likelihood of infection, the excessive and inappropriate adoption of precautionary measures [2,3] and an increased demand for health care services in a time of shortage [4]. At the other end of the spectrum, some people might deny the risks of infection and fail to engage in recommended health behaviours such as hand hygiene and social distancing [2]. A better and timely understanding of the psychological responses to infectious disease outbreaks within the community is essential for several reasons. First, the high prevalence of psychological morbidities has been documented among individuals who are directly or vicariously exposed to life-threatening situations [5,6,7]. Second, the occurrence of such psychological morbidities in a substantial proportion of the community can impact the daily functions of the affected individuals and lead to immediate social and economic consequences, such as lost job productivity and financial hardships. Third, better safeguarding of the psychological health of the community through practical mental health intervention is crucial to help prevent or ameliorate health care delivery disruptions during outbreaks [8].

Without doubt, the COVID-19 outbreak is stressful for people and communities. Fear of infection is very common during the outbreak. People were also concerned that the health care system could not cope with the COVID-19 pandemic [9]. There were not enough hospital beds and ventilators to handle the rising number of COVID-19 cases they were projected to receive. Moreover, people worried that the global economy might become worse. Fear and anxiety about the COVID-19 pandemic can be overwhelming and cause strong emotions. Besides, poor mental health during infectious disease outbreaks can be related to an individual’s misinterpretation of health-related stimuli such as bodily sensations and changes. People might misinterpret harmless bodily sensations or changes as signs of infection, causing them to become unduly distressed [2]. 

More importantly, the COVID-19 pandemic touches on fears of the 2003 severe acute respiratory syndrome (SARS) epidemic, which killed nearly 300 people in the city. As three months have passed since the first confirmed case of COVID-19 in Hong Kong, people now have a more complete picture of the extent of the pandemic. It is time to evaluate the impacts of COVID-19 on the mental health of the local people. Therefore, the aim of the present study was to evaluate the prevalence of depression and anxiety among people in Hong Kong during the COVID-19 pandemic.

## 2. Materials and Methods

### 2.1. Study Design

A cross-sectional study was conducted in Hong Kong’s general population. The data collection period was from 24 April to 3 May 2020.

### 2.2. Setting and Participants

Eligibility criteria included (i) currently living in Hong Kong, (ii) aged 18 years or older, and (iii) able to read and understand Chinese. Respondents were randomly recruited via their mobile phone numbers. A panel of mobile phone numbers were formed by an independent research company. Mobile text messages for invitation were sent to the members of the panel randomly. Once they agreed to participate in the study, we sent them a self-administered online survey link via text message or e-mail.

### 2.3. Study Instruments

The patient health questionnaire-9 (PHQ-9) was used in the present study [10]. The PHQ-9 was based on the diagnostic criteria for depression from the Diagnostic and Statistical Manual of Mental Disorders, 4th Edition (DSM-IV). The response options were: 0 = “not at all”, 1 = “several days”, 2 = “more than half the days” and 3 = “nearly every day”. A two-week recall period was used. The total score ranged from zero to 27, with a higher score indicating greater self-reported depression. A total score of ≥10 indicated possible major depression, with s a sensitivity of 80% and specificity of 92% [11,12]. The psychometric properties of the PHQ-9 have been previously confirmed in Chinese populations [10]. In the present study, the Cronbach’s alpha coefficient of the PHQ-9 was 0.92.

The generalized anxiety disorder-7 (GAD-7) was used to measure the severity of self-reported anxiety [13]. The response options were: 0 = “not at all”, 1 = “several days”, 2 = “more than half the days”, and 3 = “nearly every day”. A two-week recall period was used. The total score ranged from zero to 21, with a higher score indicating greater self-reported anxiety. For the GAD-7, a total score of ≥10 indicated possible anxiety, with the optimal point for sensitivity (89%) and specificity (82%) [14,15,16]. In the present study, the Cronbach’s alpha coefficient of the GAD-7 was 0.95.

The global rating of change scale was used to evaluate respondents’ perceived change in mental health [17]. The following single question was asked: “How would you describe your mental health now compared to the time before the COVID-19 pandemic in Hong Kong?”. The responses used a seven-point Likert scale (1 = “much worse”, 2 = “worse”, 3 = “minimally worse”, 4 = “no change”, 5 = “minimally improved”, 6 = “improved”, and 7 = “much improved”). It is suggested that the global rating of change scale has the advantages of clinical relevance, adequate reproducibility, and sensitivity to change and is easy to understand by respondents [17]. 

Respondents were also asked to respond to the following self-developed COVID-19 items: (i) I feel worried that I will be infected with COVID-19, (ii) I feel bothered because I do not have enough surgical masks, and (iii) I feel bothered because I cannot work from home. The response options and the recall period were similar to those of the PHQ-9 and the GAD-7. In addition, the respondents were asked if they had lived in Hong Kong during the 2003 SARS outbreak. 

Regarding socio-demographic factors, information on age, sex, education level, employment status, marital status, and income were also collected. 

### 2.4. Statistical Analysis

First, descriptive statistics were used to describe the socio-demographic characteristics of the respondents, the mean scores of the PHQ-9 and GAD-7, the prevalence of depression (PHQ-9 score ≥ 10) and anxiety (GAD score ≥ 10). Furthermore, the prevalence of having both depression (PHQ-9 score ≥ 10) and anxiety (GAD score ≥ 10) was also estimated. Second, multiple logistic regression models were used to explore factors associated with depression and anxiety, as well as factors associated with worsened mental health, as measured by the global rating of change scale. For the analysis, we combined the following response options:

- For worsened mental health, 1 = “much worse”, 2 = “worse”, and 3 = “minimally worse” were combined.
- For improved mental health/no change, 4 = “no change”, 5 = “minimally improved”, 6 = “improved”, and 7 = “much improved” were combined.

Adjusted odds ratios (aORs) with a 95% confidence interval (CI) were reported. The Hosmer-Lemeshow test was used to assess the model fit of the multiple logistic regressions. All statistical analyses were conducted using Statistical Package for the Social Sciences (SPSS) (version 25), with *p*-values < 0.05 indicating statistical significance. Only respondents providing full data were included in the analysis, and imputation or other substitution methods were not used.

### 2.5. Sample Size Estimation

A study in mainland China found that the prevalence of depression as measured during the COVID-19 pandemic was 48.3% [18]. Using this as our reference, at least 383 respondents were needed (confidence level = 95% and margin of error = 5%).

### 2.6. Ethics

The study protocol was approved by the institutional review board: HKU/HA HKW IRB (reference number: UW 20-217). All procedures performed in studies involving human participants were in accordance with the ethical standards of the institutional and/or national research committee and with the 1964 Helsinki declaration and its later amendments or comparable ethical standards. Electronic consent was obtained from each respondent. 

## 3. Results

In total, 774 people were approached. One hundred and sixty-three people did not respond to the invitation. One hundred and eleven people who had agreed to join the study did not answer the survey eventually. The final sample included 500 respondents, with a response rate of 64.6%. The mean age was 47.26 years (standard deviation [SD]: 15.82), 54.8% were women, 67.2% were married. 56.2% had a full-time job, 31.8% had a bachelor’s degree or above, and 32.2% had a monthly personal income > HKD$ 20,000. The mean PHQ-9 score was 5.64 (SD: 5.11). The mean GAD-7 score was 4.61 (SD: 4.45). The Pearson’s correlation coefficient between the PHQ-9 score and GAD-7 score was 0.86 (*p*-value < 0.001). The prevalence rate of mild depression (PHQ-9 score 5–9) was 29.00%, 12.80% for moderate depression (PHQ-9 score 10–14), 6.20% for moderately severe depression (PHQ-9 score 15–19), and 0.80% for severe depression (PHQ-9 score 20–27). 

The prevalence rates of depression (PHQ-9 score ≥ 10) and anxiety (GAD-7 score ≥ 10) were 19.8% and 14.0%, respectively. The prevalence rate of having both depression and anxiety was 12.40%. In addition, 25.4% of the respondents reported that their mental health had deteriorated since the COVID-19 pandemic began. Table 1 shows the respondents’ characteristics and the depression and anxiety prevalence rates. 

Multiple logistic regression models controlling for age, sex, education level, employment status, marital status, and income revealed that individuals who were not living in Hong Kong during the 2003 SARS outbreak were more likely to have depression (aOR = 2.78; 95% CI, 1.14–6.79, *p*-value = 0.024) than those who lived in Hong Kong during that period. Meanwhile, individuals who were more worried about being infected by COVID-19 were more likely to have depression (aOR = 1.86; 95% CI, 1.37–2.52, *p*-value <0.001), anxiety (aOR = 1.73; 95% CI, 1.25–2.40, *p*-value = 0.001), combined depression and anxiety (aOR = 1.80; 95% CI, 1.28–2.53, *p*-value <0.001), and worsened mental health (aOR = 1.94; 95% CI, 1.48–2.55, *p*-value <0.001). Individuals who were more bothered by having not enough surgical masks were more likely to have depression (aOR = 1.44; 95% CI, 1.08–1.91, *p*-value = 0.012), anxiety (aOR = 1.51; 95% CI, 1.12–2.04, *p*-value = 0.007), combined depression and anxiety (aOR = 1.49; 95% CI, 1.08–2.04, *p*-value = 0.014), and worsened mental health (aOR = 1.36; 95% CI, 1.05–1.75, *p*-value = 0.018). Individuals who were more bothered by not being able to work from home were more likely to have depression (aOR = 1.46; 95% CI, 1.18–1.82, *p*-value = 0.001) and anxiety (aOR = 1.32; 95% CI, 1.04–1.68, *p*-value = 0.023) and combined depression and anxiety (aOR = 1.34; 95% CI, 1.04–1.72, *p*-value = 0.023). Table 2 shows the results of the simple logistic regression and multiple logistic regression analyses. 

## 4. Discussion

To the best of our knowledge, this is the first population study to evaluate the depression and anxiety of people in Hong Kong during the COVID-19 pandemic. The study provides important and timely data about the impact of COVID-19 on individuals’ mental health. 

In the present study, 19% of the respondents had depression (PHQ-9 score ≥10) and 14% had anxiety (GAD score ≥10). The prevalence of depression was much higher than that reported in previous studies [19]. For example, a meta-analysis that evaluated the aggregate prevalence of depression in communities from multiple countries between 1994 and 2014 reported that the lifetime prevalence of depression was 10.8%. [19]. Moreover, it is noteworthy that the prevalence of depression found in the present study is higher than that found among health workers in Wuhan, mainland China [20]. The study found that the prevalence of depression (PHQ-9 score ≥10) was 13.5% [20]. In addition, a meta-analysis of the prevalence of anxiety disorders in mainland China from 2000 to 2015 reported that the lifetime prevalence of generalised anxiety disorder was 4.66% [21]. Compared with previous studies in Hong Kong, the prevalence rates of depression (19.8% vs 10.7%) [22] and anxiety (14.0% vs 4.1%) [23] were much higher in the presence of the pandemic. Moreover, 25.4% of the respondents reported that their mental health had deteriorated since the pandemic began. This alarming finding suggests that the pandemic has substantially affected the mental health of people in Hong Kong. High anxiety during the pandemic is problematic because a recent study found that coronavirus-related anxiety was strongly associated with functional impairments, alcohol or drug coping, negative religious coping, extreme hopelessness, and passive suicidal ideation [24]. Besides, our findings are also consistent with previous studies which found that exposing public health emergency [25] such as Ebola outbreak [26], earthquakes [27], and SARS [28] can cause mental health issues. 

In response to the current COVID-19 crisis, the local government requested the closures of school and non-essential business, banned large gatherings, requested quarantines for people travelling from abroad, and encouraged social distancing. As such, people have had to stay at home for most of the day throughout the COVID-19 pandemic period. Given this situation, it is important to note that a systematic review reported consistent evidence linking social isolation and loneliness to poor mental health [29]. Another review reported that quarantines during disease outbreaks could lead to poor mental health outcomes due to frustration, boredom, inadequate basic supplies and inadequate information [30]. 

Another possible explanation of the poorer mental health during the COVID-19 is related to COVID-19 information overload which has been characterized by contradictory information from different international and local authorities, experts, and scientists with different backgrounds, and mass media [31]. Social media, such as Twitter and Facebook, is commonly used to update and obtain latest information of COVID-19. However, people have been overwhelmed by receiving too much information of COVID-19 [32]. A recent study in mainland China found that a higher frequency of social media exposure increased the likelihood of having anxiety as measured by the GAD-7 [18]. In fact, previous studies found that indirect exposure to mass trauma through the media could lead to post-traumatic stress disorder [33]. Furthermore, a previous study in South Korea also found that social media exposure was positively related to forming risk perceptions during Middle East respiratory syndrome coronavirus outbreak [34]. Social media is a double-edged sword. They can rapidly disseminate important information so that people can adopt appropriate public measure to protect themselves. However, rumours, misinformation, and fear can also readily spread through social media, further heightening fear and anxiety [2].

As expected, people who were more worried about being infected with COVID-19 were more likely to have poor mental health. During a pandemic, people are fearful that they or their family members will fall ill and are very uncertain of the repercussion of the pandemic. Furthermore, discrimination and stigma related to infectious diseases might make people fearful of infection, which can also affect their mental health status [35]. Recent studies among Italian [36] and Iranian [37] populations found that fear of COVID-19 was significantly correlated with depression and anxiety, as measured by the hospital anxiety and depression scale; the authors also explained that fear of COVID-19 may be exacerbated by coexisting depression and anxiety disorders [36]. 

We found that respondents who were more bothered by not having enough surgical masks were more likely to have poor mental health. After the SARS outbreak, people in Hong Kong realised the importance of wearing surgical masks to prevent the transmission of respiratory viruses from ill persons. However, at the beginning of the COVID-19 pandemic, people in Hong Kong faced shortages, rising prices and increasingly frantic quests for surgical masks. Many people in Hong Kong queued outside pharmacies and shops every day hoping to stock up on surgical masks, but their attempts were always in vain. Without doubt, the shortage of surgical masks in Hong Kong and uncertainty of when and where surgical masks would become available have made people in Hong Kong feel worried and anxious. 

Personality traits should also be considered when we would like to understand individuals’ reactions to pandemics [2]. It is suggested that some personality traits such as neuroticism are associated with the proneness to experience negative emotions in response to psychosocial stressors. First, individuals who score high on neuroticism are more likely to experience anxiety, worry, fear, depression, and loneliness [2]. Those who score high on neuroticism also tend to misinterpret bodily sensations as indications of serious disease. The severity of an individual’s neuroticism predicts their likelihood of being distressed by the threat of infection. For example, a study in Taiwan found that neuroticism influenced the mental health of health care workers during the SARS outbreak [2,38]. Second, people who score high on trait anxiety tend to view the world as dangerous and threatening. A previous study in Hong Kong found that trait anxiety predicted the level of SARS-related anxiety the SARS outbreak [2,39]. Third, people who score high on the overestimation of threat tend to overestimate the badness and likelihood of adverse events and see themselves as being particularly vulnerable to threats [2]. Previous studies have found that the overestimation of threat predicts anxiety in response to the outbreak of infectious diseases such as avian flu, Ebola virus, SARS, and Swine flu [2]. In future studies, personal traits should also be measured to further explore the individual difference in response to infectious outbreaks. 

One important finding of the present study was that people who were bothered by not being able to work from home were more likely to have depression and anxiety than others. During the COVID-19 pandemic, people in Hong Kong tend to stay at home to minimize the risk of infection. Although the Hong Kong government appealed to companies to institute work from home arrangements, the decision to implement this practice was entirely up to the employers. It is understandable that people who were not able to work from home were depressed and anxious when the number of COVID-19 cases in Hong Kong kept increasing. Moreover, people worried that riding on public transport would make them more susceptible to the disease. Therefore, we recommend that people who are not able to work from home receive more attention during the pandemic. For example, protective equipment, such as surgical masks and hand sanitizers, should be provided to them for protection and to ease their anxiety. 

Another noteworthy finding was that people who were not living in Hong Kong during the 2003 SARS outbreak were more likely to have depression, suggesting that the first experience of an infectious disease outbreak is an incredibly stressful event. In contrast, individuals who experienced SARS in 2003 might have more psychological preparation to fight the current pandemic. People in Hong Kong who experienced SARS probably already knew what they should do to protect themselves from the current outbreak, such as practicing hand hygiene, wearing surgical masks and engaging in social distancing [40]. Qualitative studies should be conducted to understand how exposure to the SARS outbreak in 2003 has helped people in Hong Kong cope with the COVID-19 pandemic. 

Similar to the emotional effects of other stressors, pandemic-related distress may fade without any intervention. However, for people who experience severe levels of pandemic-related distress, more intensive interventions should be provided. For example, people with major depressive disorder or post-traumatic stress disorder triggered by the loss of loved ones or other traumatic events, and people with generalized anxiety disorder that is triggered by the uncertainty associated with a pandemic should be prioritised to receive psychosocial interventions such as cognitive behavioural therapies [2]. Besides, when we implement mental health programmes during pandemics, it is important that such programme will not increase the burden on healthcare providers and the risk of infection being spread to others [2].

The current study provides the preliminary data about the impact of COVID-19 on mental health. Longitudinal studies are needed to understand the trajectories of mental health during the pandemic of COVID-19. Cross-cultural studies should also be considered to explore the regional variation of depression and anxiety during the COVID-19 pandemic. Last but not least, qualitative studies are needed to understand how people cope with the pandemic and what psychosocial supports they need during the pandemic. The data are very important to future pandemic management. 

### Limitations

There are some limitations that should be considered when interpreting this study’s findings. First, all of the outcomes were self-reported, which might lead to recall bias. However, using self-reported scales to measure depression and anxiety is common because of its convenience and low cost. Second, since a self-administered online questionnaire was used in the study, the computer literacy of respondents might have affected how they responded to the questionnaire. However, given the COVID-19 pandemic, a household survey was not considered appropriate. Third, this was a cross-sectional study, so the temporal change and trajectory of the respondents’ mental health could not be observed. Fourth, the study findings in the present study might not be generalisable to other populations. Factors such as the prevalence of the COVID-19 and different mortality rate might affect the impacts of COVID-19 on mental health. Fifth, we did not collect data about pre-existing diagnoses of depression and anxiety. Thus, we were not able to control for them in the analysis. Sixth, potential self-selection bias inherent in the study should be noted. It was possible that individuals who were more concerned about their mental health were more likely to join the study. 

## 5. Conclusions

Nineteen per cent of the respondents had depression and fourteen per cent had anxiety during the COVID-19 pandemic. Our findings suggested that COVID-19 has substantially affected individuals’ mental health. Furthermore, people who did not experience the SARS outbreak, people who were more worried about being infected by COVID-19, people who were more bothered by having not enough surgical masks and people who were more bothered by not being able to work from home had a poorer mental health status. One key policy implication of the present study is that governments should provide psychological support to citizens during a pandemic. For example, brief, home-based psychological interventions should be developed to diminish the adverse impacts of COVID-19 on mental health. 

## Figures and Tables

**Table 1:** Demographics of the respondents.

**Mean age (SD)**	47.26 (15.82)
	N (%)
**Sex**	
Male	226 (45.20)
Female	274 (54.80)
**Marital status**	
Not currently married	164 (32.80)
Currently married	336 (67.20)
**Education status**	
Below bachelor’s degree	341 (68.20)
Bachelor’s degree or above	159 (31.80)
**Employment status**	
Not having a full-time job	219 (43.80)
Having a full-time job	281 (56.20)
**Monthly personal income**	
HKD$ 20,000 or below	339 (67.80)
HKD$ 20,001 or above	161 (32.20)
**Depression (PHQ-9)**	
Mean PHQ-9 score (SD)	5.64 (5.11)
PHQ-9 score 0–4 (none)	256 (51.20)
PHQ-9 score 5–9 (mild)	145 (29.00)
PHQ-9 score 10–14 (moderate)	64 (12.80)
PHQ-9 score 15–19 (moderately severe)	31 (6.20)
PHQ-9 score 20–27 (severe)	4 (0.80)
PHQ-9 score ≥ 10	99 (19.80)
**Anxiety (GAD-7)**	
Mean GAD-7 score (SD)	4.61 (4.45)
GAD-7 score < 10	430 (86.00)
GAD-7 score ≥ 10	70 (14.00)
**Depression and Anxiety**	
PHQ-9 score ≥ 10 and GAD-7 score ≥ 10	62 (12.40)
**Global rating of change scale**	
Improved mental health/no change	372 (74.40)
Worsened mental health	127 (25.40)
Missing value	1 (0.20)

Abbreviations: PHQ-9: patient health questionnaire-9; GAD-7: generalized anxiety disorder-7; SD: standard deviation; HKD: Hong Kong dollar.

**Table 2:** Logistic regression to explore factors associated with poor mental health.

	**Depression**			**Depression ^1^**		
	**Crude OR**	**95% CI**	***p*-Value**	**aOR**	**95% CI**	***p*-Value**
Worried about being infected by COVID-19	2.17	(1.68, 2.81)	<0.001	1.86	(1.37, 2.52)	<0.001
Bothered by having not enough surgical masks	1.90	(1.50, 2.39)	<0.001	1.44	(1.08, 1.91)	0.012
Bothered by not being able to work from home	1.46	(1.21, 1.76)	<0.001	1.46	(1.18, 1.82)	0.001
Not living in Hong Kong during the 2003 SARS	4.24	(1.97, 9.11)	<0.001	2.78	(1.14, 6.79)	0.024
	**Anxiety**			**Anxiety ^2^**		
	**Crude OR**	**95% CI**	***p*-Value**	**aOR**	**95% CI**	***p*-Value**
Worried about being infected by COVID-19	2.15	(1.61, 2.86)	<0.001	1.73	(1.25, 2.40)	0.001
Bothered by having not enough surgical masks	1.96	(1.52, 2.53)	<0.001	1.51	(1.12, 2.04)	0.007
Bothered by not being able to work from home	1.39	(1.12, 1.71)	0.002	1.32	(1.04, 1.68)	0.023
Not living in Hong Kong during the 2003 SARS	3.61	(1.60, 8.12)	0.002	2.38	(0.97, 5.88)	0.059
	**Combined Depression and Anxiety**			**Combined Depression and Anxiety ^3^**		
	**Crude OR**	**95% CI**	***p*-Value**	**aOR**	**95% CI**	***p*-Value**
Worried about being infected by COVID-19	2.20	(1.63, 2.97)	<0.001	1.80	(1.28, 2.53)	0.001
Bothered by having not enough surgical masks	1.96	(1.50, 2.56)	<0.001	1.49	(1.08, 2.04)	0.014
Bothered by not being able to work from home	1.41	(1.13, 1.77)	0.002	1.34	(1.04, 1.72)	0.023
Not living in Hong Kong during the 2003 SARS	3.55	(1.54, 8.20)	0.003	2.39	(0.93, 6.14)	0.070
	**Worsened Mental Health**			**Worsened Mental Health ^4^**		
	**Crude OR**	**95% CI**	***p*-Value**	**aOR**	**95% CI**	***p*-Value**
Worried about being infected by COVID-19	2.22	(1.74, 2.85)	<0.001	1.94	(1.48, 2.55)	<0.001
Bothered by having not enough surgical masks	1.75	(1.40, 2.17)	<0.001	1.36	(1.05, 1.75)	0.018
Bothered by not being able to work from home	1.14	(0.95, 1.36)	0.158			
Not living in Hong Kong during the 2003 SARS	0.93	(0.39, 2.23)	0.867			

^1^ Hosmer and Lemeshow Test: chi-square: 11.048; degrees of freedom: 8; *p*-value: 0.199. ^2^ Hosmer and Lemeshow Test: chi-square: 8.520; degrees of freedom: 8; *p*-value: 0.384. ^3^ Hosmer and Lemeshow Test: chi-square: 5.453; degrees of freedom: 8; *p*-value: 0.708. ^4^ Hosmer and Lemeshow Test: chi-square: 8.453; degrees of freedom: 8; *p*-value: 0.390. All multiple logistic regression models were controlled for age, gender, marital status, education level, employment status, and income level. Abbreviations: aOR: adjusted odds ratio; CI: confidence interval.
